# Heterocycles in Medicinal Chemistry II

**DOI:** 10.3390/molecules29204810

**Published:** 2024-10-11

**Authors:** Josef Jampilek

**Affiliations:** 1 Department of Chemical Biology, Faculty of Science, Palacky University Olomouc, Slechtitelu 27, 783 71 Olomouc, Czech Republic; josef.jampilek@gmail.com; 2 Department of Analytical Chemistry, Faculty of Natural Sciences, Comenius University, Ilkovicova 6, 842 15 Bratislava, Slovakia

Carbon has a unique position among the elements, due to the fact that its valence shell has four electrons and is therefore quadrivalent in the excited state. Carbon atoms form strong covalent bonds and can also join together to form long chains or rings [1]. The most basic division of all organic compounds can be according to the representation of individual elements. One group contains only the “backbone” composed of carbons and hydrogens, while the second, much more comprehensive group also includes other atoms, so-called heteroatoms. Most often, the term “heterocyclic compounds” in the abbreviation “heterocycles” refers to organic cyclic substances that, in addition to carbon atoms, also have heteroatoms in the cycle. According to the type of heteroatom, these compounds can be divided into nitrogen, oxygen, and sulfur, but they can also contain other heteroatoms, such as selenium, phosphorus, iron, etc. [2,3]. According to the number of heteroatoms, they are divided into monovalent, divalent, and polyvalent. As cyclic hydrocarbons, heterocycles are most commonly five- and six-membered and may be saturated or unsaturated; depending on the structure and the actual type and number of heteroatoms, they may show signs of aromaticity. Heterocycles can combine to form various fused systems, e.g., with a benzene ring or with other heterocycles [1,2,3].

By introducing a heteroatom into the carbon backbone, the compound acquires completely different, often unique properties (reactivity, lipo-hydrophilic properties, polarity, or ability to interact with the environment) from the parent hydrocarbons [2,4,5,6,7,8,9,10,11,12,13,14,15]. Heterocycles thus form the most important traditional branch of organic chemistry, and research interest in heterocycles is growing due to their biological (medical) and industrial applications [4,5,6,7,8,9,10,11,12,13,14,15,16,17,18,19,20], as documented in Figure 1. Heterocycles are found in natural substances, and some are necessary for life (e.g., purine/pyrimidine backbones of DNA/RNA, building blocks of chlorophyll, hemoglobin, essential amino acids in proteins, vitamins, etc. [4,20,21,22,23]).

From the point of view of significance, it does not matter whether they are isosterically or bioisosterically substituted carbons or carbon substructures. Most biologically active chemical entities contain a heterocycle [4,19,24]. This fact reflects the central role of heterocycles in the modern design of drugs or even agrochemicals [4,5,18,19,21,24,25,26,27,28,29,30,31]. Many heterocyclic scaffolds can be considered privileged structures. There is no therapeutic group of drugs that does not contain heterocyclic drugs, so it can be stated that they have a wide spectrum of biological activities [19,24,26,27,31]. Therefore, heterocycles are of fundamental importance to medicinal chemists, as their use can expand the chemical universe from which to draw new compounds for biomedical research and streamline drug discovery programs. Historically, many procedures have been developed for the targeted synthesis of heterocycles (target-oriented synthesis or diversity-oriented synthesis), although there is still the possibility of their isolation from natural sources and their subsequent modification [2,3,32,33,34,35,36,37,38].

The articles in this Special Issue provide some overview of recent developments in the field of heterocycle research. The study of drug lipophilicity combining experimental and chemometric methods (principal component analysis and cluster analysis) and comparison of chromatographically obtained and theoretical lipophilicity descriptors represents a useful concept for simplifying the prediction of drug-like properties of molecules [39]. Many heterocycles have the potential to act as anti-infective agents. Ring-substituted pyrazoles have been tested as potential agricultural fungicides against a number of phytopathogenic fungi, and some of the listed compounds showed activity comparable to pyraclostrobin [40]. The cyclic analogues of pyrazoles—a series of twenty-two indazole derivatives studied as antiprotozoa against *Entamoeba histolytica*, *Giardia intestinalis,* and *Trichomonas vaginalis*—appear to hold great promise as potential therapeutics [41]. Ahmad et al. prepared a series of substituted *N*-(4-methylpyridin-2-yl)- thiophene-2-carboxamides showing high activity against pathogenic extended-spectrum β-lactamase producing *Escherichia coli*, which, in addition to promising efficacy, have the ability to inhibit β-lactamases as well [42]. A series of new *N*-substituted benzimidazole carboxamides were evaluated for their overall anti-invasive activity, i.e., both antimicrobial (Gram-positive bacteria) and anticancer (H460, HCT 116, MCF-7, HEK293) efficacies [43]. Synthetic compounds (nitroimidazoles, 5-(indol-2-yl)pyrazolo[3,4-*b*]pyridines) [44,45] or semi-synthetic derivatives (steviol or isosteviol) [46] have been evaluated for their ability to eliminate cancer cells and prevent the emergence of cancer lines resistant to clinically used drugs. The above-mentioned indications of heterocyclic derivatives aimed at anti-infective agents and anticancer drugs are complemented by the following two publications: Zolotareva et al. provide a review of potential antidiabetic agents based on three heterocyclic compounds, namely morpholine, piperazine, and piperidine [47]; and Kim et al. investigated benzylidene-3-methyl-2-thioxothiazolidin-4-ones with antioxidant activities as potential tyrosinase inhibitors for the treatment of hyperpigmentation-related disorders [48].

## Figures and Tables

**Figure 1:**
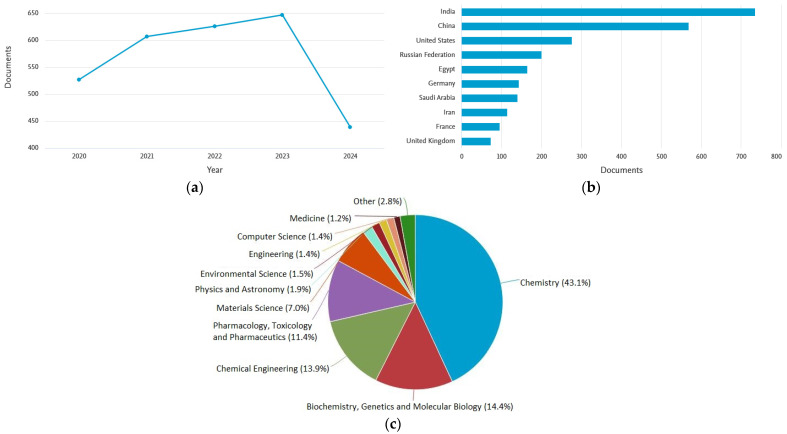
Number of publications according to Scopus database published in the years 2020–2024 (as of 30 September 2024) containing in the title of the article, the abstract or the keywords, “heterocycle” and “heterocyclic compounds” (**a**), publications by country (**b**), publications by subject areas (**c**).
